# Bibliometric analysis of the top-cited articles on islet transplantation

**DOI:** 10.1097/MD.0000000000008247

**Published:** 2017-11-03

**Authors:** Qiang-Hong Pu, Qiu-Ju Lyu, Huan Liu, Kai-Hua Fan

**Affiliations:** a Department of Pharmacy; b Department of Endocrinology, People's Hospital of Leshan, Sichuan, P.R. China.

**Keywords:** bibliometrics, citation analysis, citation classics, islet transplantation

## Abstract

**Aims:**

To identify and characterize the top-cited articles in the field of islet transplantation.

**Methods:**

We used the Science Citation Index Expanded database to identify the most frequently cited articles published after 1900. Articles were evaluated using the following characteristics: citation number, publication year, study design, references, country and institution of origin, authorship, and journal. Keyword analysis and citation networks were used to analyze research trends.

**Results:**

The most frequently cited articles received between 146 and 2988 citations; the median was 291. All of the most frequently cited articles were published between 1972 and 2012, and 85 articles were published after 1990. The most popular study design involved basic science (75 articles). The leading countries were the United States (US) and Canada, and the leading institutions were the University of Alberta, Canada, and the University of Minnesota, in the US. Journals specializing in diabetes or transplantation published more than half of the articles (n = 53, 52%), with the journal *Diabetes* publishing the largest number (n = 30). No association was found between a journal's impact factor and the number of top-cited articles it published. There was no correlation between the number of citations and the number of years since publication, authors, participating institutions, or countries involved. Top-cited articles focused on 2 themes: the use of antirejection immunotherapy or biocompatible encapsulations to prolong graft survival, and assessments of the efficacy of islet transplants, in particular, islet allografts.

**Conclusions:**

Our study can help researchers to identify and decipher the characteristics of top-cited articles in the field of islet transplantation. Just as clinically successful allografts are carried out using the Edmonton protocol, autografts and xenografts should be similarly strengthened to solve problems relating to immune rejection and islet sources, respectively.

## 1 Introduction

Diabetes now affects 382 million people and causes approximately 4.6 million deaths every year globally.^[1,2]^ It is also a major risk factor for cardiovascular disease, stroke, renal failure, blindness, and lower limb amputation. In various treatments for diabetes, islet transplantation may be the most promising strategy for clinically curing this condition, particularly in the case of type 1 diabetes mellitus.^[3,4]^ Many articles on islet transplantation have been published during the past decades, and the number continues to increase. Top-cited articles (also called classic articles or citation classics) in the field of islet transplantation have not been identified or characterized, despite their importance in promoting the development of islet transplantation.

The number of citations an article receives after publication is an important measure of its significance within the scientific community, because the influence of a paper in a particular discipline is usually proportional to the number of citations it receives.^[5]^ Citation analysis is therefore one method of bibliometric analysis that has been used to quantify the relative importance of a scientific paper by examining the citations attributed to that paper.^[6,7]^ Recently, top-cited articles have been identified using citation analysis in many medical fields, including head and neck surgery, lumbar spine surgery, plastic surgery, orthopedic elbow surgery, neurosurgery, hand surgery, diabetes, and hypertension.^[8–17]^ As far as we know, this type of identification has not been used in the field of islet transplantation. In this bibliometric study, we have carried out a citation analysis to identify and characterize the 100 most frequently cited articles on islet transplantation.

## 2 Methods

### 2.1 Study design and data search

We used the Science Citation Index Expanded (SCIE, 1900–2017) database to identify the 100 most frequently cited articles in islet transplantation research because this platform has been used to identify top-cited articles in many other medical specialties.^[10,14,15,18]^ Search expressions included the following terms and Boolean operators: “transplant∗” OR “graft∗” OR “autotransplant∗” OR “autograft∗” OR “isotransplant∗” OR “isograft∗” OR “allotransplant∗” OR “allograft∗” OR “homotransplant∗” OR “homograft∗” OR “heterotransplant∗” OR “heterograft∗” OR “xenotransplant∗” OR “xenograft∗”. These were used to retrieve the transplantation research. To identify studies about islets, the Boolean operator “AND” was added to the transplantation-specific terms, followed by the search terms (“islet∗”OR (“island∗” and “pancrea∗”) OR (“island∗” and “Langerhans”)). The search, performed on May 7, 2017, yielded a complete list of articles on islet transplantation. No language was restricted in this search. Ethical approval was not required in this study, because no patients were enrolled.

### 2.2 Inclusion and exclusion criteria

The inclusion criteria were as follows: (1) papers focusing on islet transplantation as the main topic; (2) original articles and reviews. The exclusion criteria were as follows: (1) articles focused on broad areas, with no emphasis on islet transplantation; (2) articles focused on transplantations of other organs or cells, such as heart, liver, kidney, lung, or stem cell transplants; (3) meeting abstracts, letters, editorial materials, corrections, book chapters, errata, discussions, and book reviews.

### 2.3 Identification and assessment of the top 100 articles

To ensure a consistency of data abstraction, we used the method developed by Lim et al.^[19]^ Three investigators (QHP, QJL, and HL), including 1 clinical pharmacist and 2 physicians in the field of endocrinology (with 10, 11, and 3 years of experience, respectively), initially reviewed the same 150 articles on islet transplantation which were randomly chosen. Any disagreements were resolved in a consensus meeting consisting of 4 authors (QHP, QJL, HL, and KHF). Although no formal interobserver reliability testing was conducted between the investigators, the disagreements were even rare. After initial pilot training, the searched results were manually assessed by 3 independent authors (QHP, QJL, and HL).

The search results were arranged from most to least citations using the “times cited” option. Later, the results were assessed by 3 independent authors (QHP, QJL, and HL) to compile 1 comprehensive list of the 100 most frequently cited articles. Finally, the top 100 articles were reviewed and data were extracted, in accordance with the method developed by Azer and Azer.^[20]^

The 100 selected articles were analyzed using the following parameters: citation number, publication year, study design, references, country and institution of origin, authorship, and journal. Citation numbers for the top 100 articles were obtained by searching the SCOPUS database. Numbers listed only in the SCIE database were not included. In addition to a randomized controlled trial (RCT), this study incorporated other study designs, such as nonrandomized controlled and uncontrolled trials, including cohort, case-control, and cross-sectional studies, and a case series. Articles were grouped into the following six6 categories by study design and goal: (1) a RCT; (2) an observational study including cohort, case-control, and cross-sectional studies, and a case series; (3) basic science, including bench-top laboratory research and research involving animal or cell models; (4) a review; (5) a meta-analysis; and (6) clinical guidelines. Once a list of journal impact factors had been obtained from the Journal Citation Report 2016, we studied the correlation between each journal's impact factor and the number of top 100 articles it published. We also investigated the correlation between the number of citations and other characteristics, namely, the number of years since publication, and the number of authors, participating countries, and institutions involved. SCIE reference data were used to complete the reference analysis. Because keywords were separated into different time periods to demonstrate trend variations on research topics in the fields of lung cancer and obstructive sleep apnea, the combined analysis of author keywords and keywords plus in SCIE database demonstrated research trends in islet transplantation. ^[21,22]^ To visualize the citation links and research topics of the 100 most frequently cited articles, we used CitNetExplorer Software version 1.0 to carry out a citation network analysis, using Kusumastuti et al's^[23]^ method. Lastly, we chose Oxford Centre for Evidence-based Medicine—Levels of Evidence (2009 edition) to assess the top-cited articles.

### 2.4 Statistical analyses

All data were analyzed using GraphPad Prism software (version 6, GraphPad Software Inc. CA). Nonparametric Spearman rank correlations were used to determine the correlations among variables. All statistical tests were 2-tailed; a *P* value of <.05 was considered statistically significant.

## 3 Results

### 3.1 Citations

Table 1     shows the top-cited articles in islet transplantation research in descending order, according to total citations. Two other articles had the same number of citations as the paper ranked 100, bringing the total number of articles to 102. The median number of citations for the top 100 articles was 291 (range 146–2988). We found a generally positive correlation between the total number of citations in SCIE, citation density (citations per year), and citations in SCOPUS (*r* = 0.63, *P* < .05; *r* = 0.82, *P* < .05). The same article had the highest number of citations in SCIE and SCOPUS, and the highest citation density: a clinical trial about islet transplantation in seven patients with type 1 diabetes mellitus using glucocorticoid-free immunosuppressive regimen, published in the *New England Journal of Medicine* in 2000 by Shapiro et al.^[24]^ All articles were published in the English language. We also analyzed references in the top 100 articles using SCIE data. The reference numbers for these studies ranged from 4 to 180, with an average of 39 references. Further analysis demonstrated that 9 references were high-frequency (≥10 times) (Table 2); 5 of these were included in the top 100 articles. Interestingly, the article published in the *New England Journal of Medicine* in 2000 by Shapiro et al^[24]^ was still ranked number 1 (having been cited 23 times). In addition, 4 high-frequency references were related to the method of islet isolation.

**Table 1:**
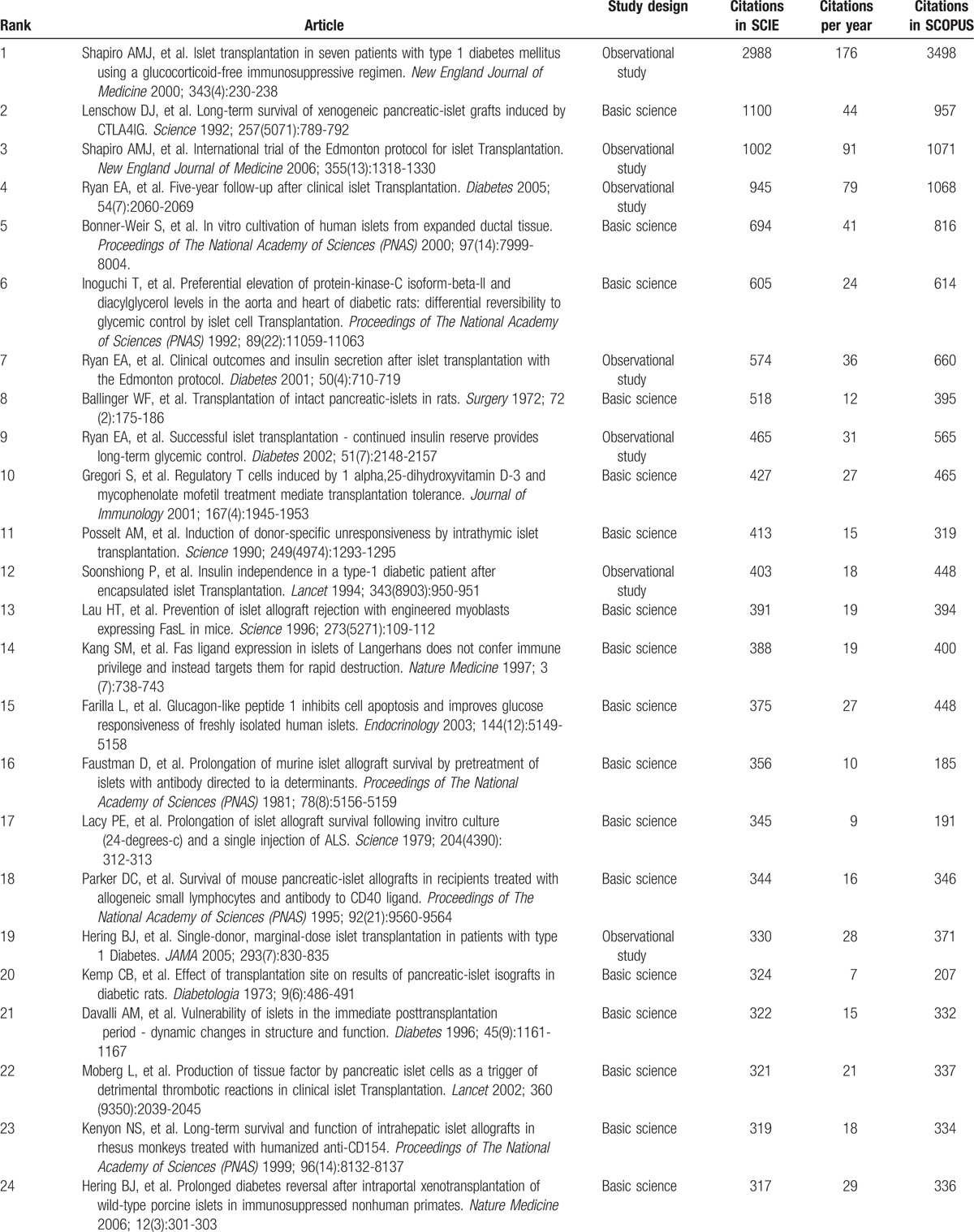
The top-cited articles in islet transplantation research.

**Table 1 (Continued):**
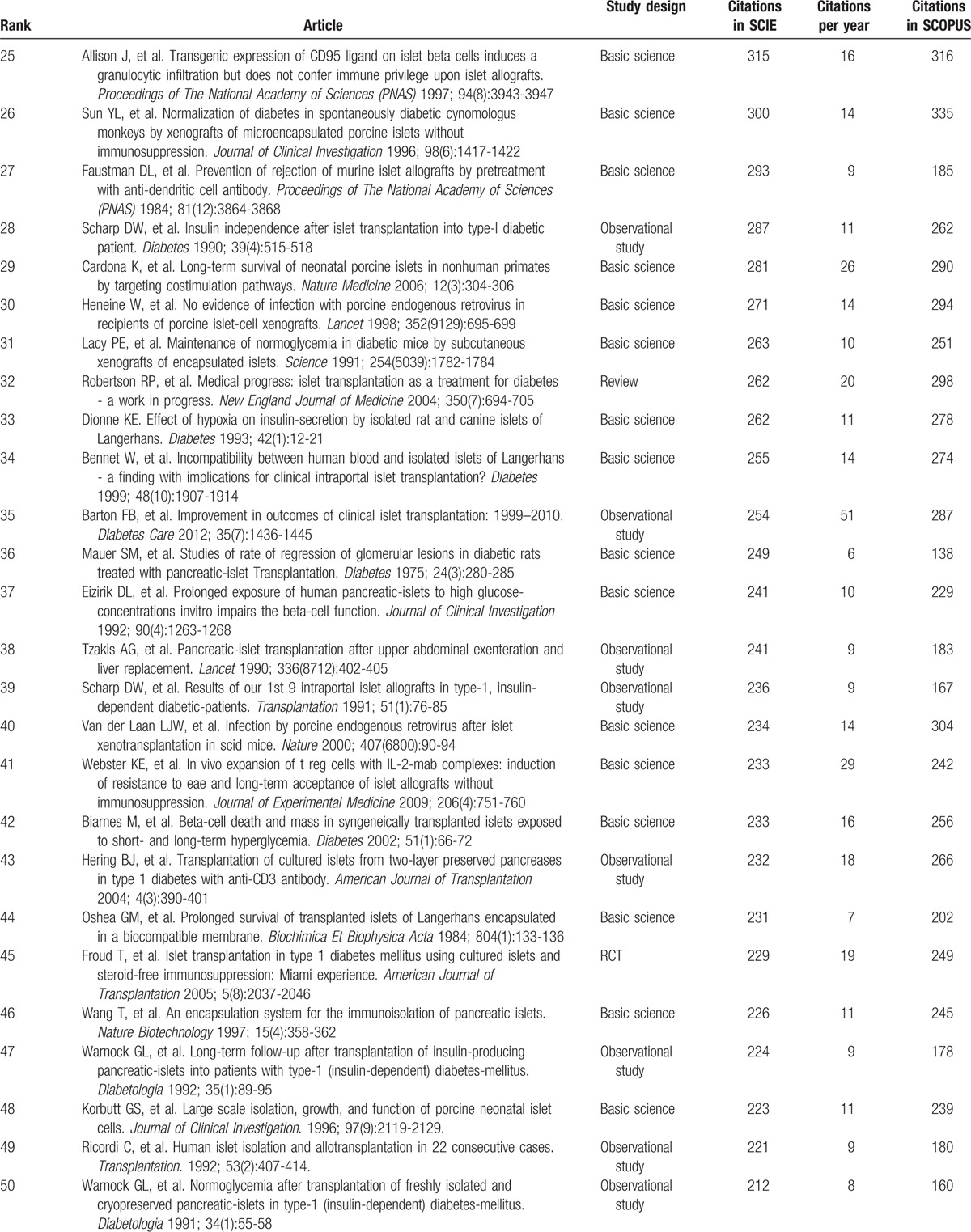
The top-cited articles in islet transplantation research.

**Table 1 (Continued):**
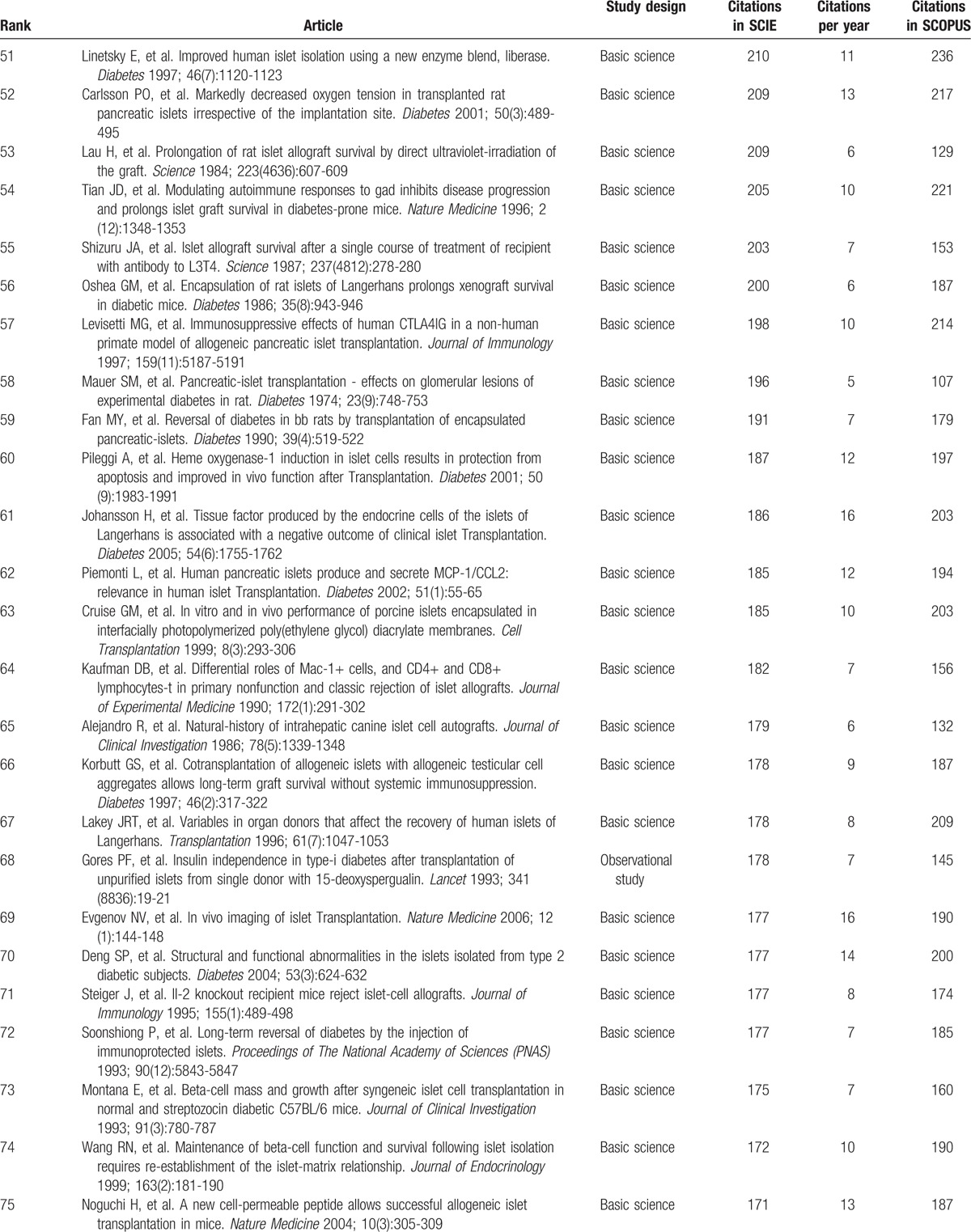
The top-cited articles in islet transplantation research.

**Table 1 (Continued):**
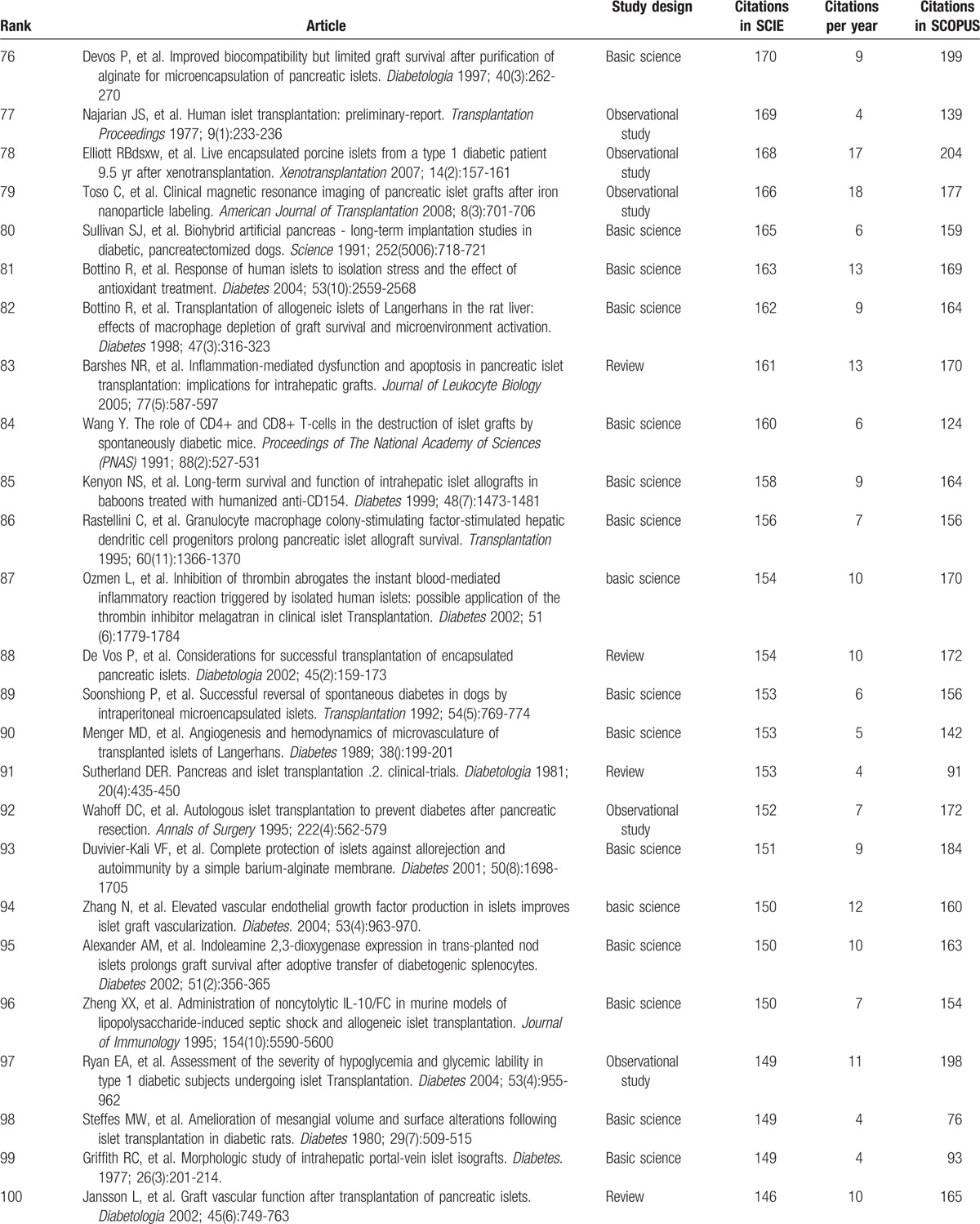
The top-cited articles in islet transplantation research.

**Table 1 (Continued):**
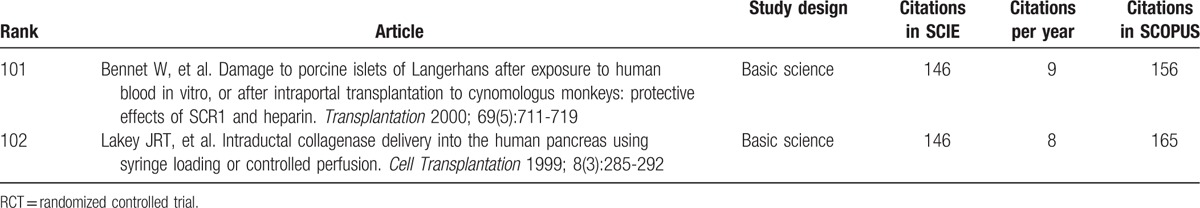
The top-cited articles in islet transplantation research.

**Table 2:**
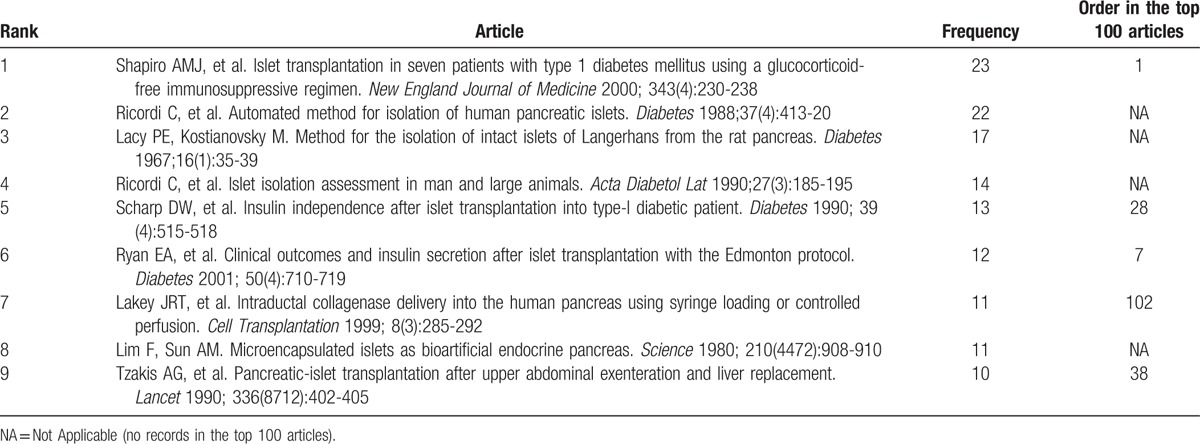
High-frequency references in the top-cited articles.

### 3.2 Publication year, study design, and levels of evidence of the top-cited articles

**Figure 1:**
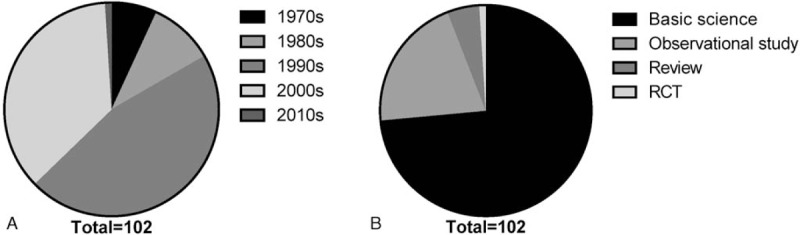
Distribution of top-cited articles in the field of islet transplantation. (A) Time distribution by decade. The results show that the largest cluster of articles (47) was published during the 1990s. (B) Type distribution by study design. Observational studies predominated among top-cited articles.

These articles were published over a 40-year period (from 1972 to 2012). Figure 1A shows the distribution of top-cited articles by decade of publication. The largest group of articles (47) was published in the 1990s; overall, 85 articles were published after 1990. Figure 1B summarizes the study design of the top-cited articles. Most of these articles were in basic science (n = 75), whereas the remaining articles were observational studies (n = 21), reviews (n = 5), and RCTs (n = 1). No articles involved guidelines or meta-analyses.

Oxford Centre for Evidence-based Medicine—Levels of Evidence (2009 edition) was used to evaluate all studies. There was only 1 study at levels 1c, and 21 and 80 studies at levels 4 and 5, respectively.

### 3.3 Authors, countries, and institutions

We carefully analyzed the distribution of primary authors of top-cited articles. Fifteen of the authors had published more than 1 article, as shown in Table 3. Ryan et al published the most (n = 4), followed by Hering et al and Soonshiong et al (n = 3).

**Table 3:**
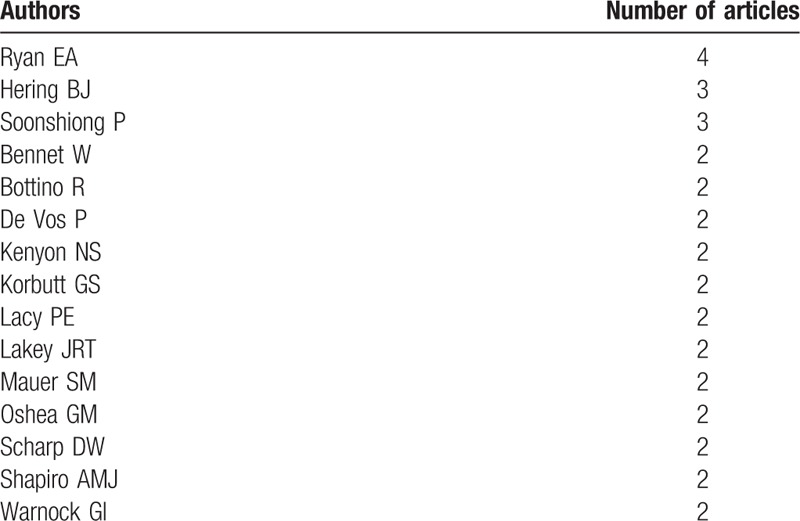
Primary authors of top-cited articles on islet transplantation.

**Figure 2:**
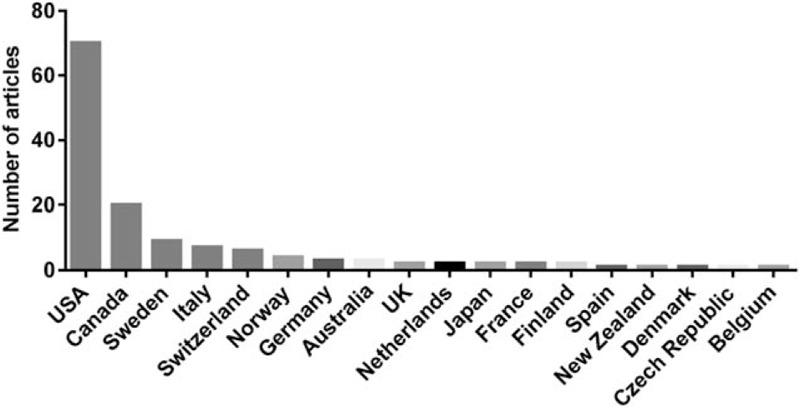
Countries of origin of top-cited articles in the field of islet transplantation.

The top-cited articles originated from 18 countries (Fig. 2). The countries producing the most articles were the USA (n = 70), followed by Canada (n = 20), Sweden (n = 9), and Italy (n = 7). Only 23 articles were written by multinational collaborations; 79 articles were authored by researchers from the same country.

Sixteen institutions published 3 or more of the 100 most frequently cited articles (Table 4). Ten (62.5%) of these institutions were located in the US. Of the 8 (37.5%) institutions outside the US, 4 were in Canada (n = 2) and Sweden (n = 2), and 2 were in Italy (n = 1) and Switzerland (n = 1). The institutions producing the most articles were the University of Alberta (n = 15), followed by the University of Minnesota (n = 14), Harvard University (n = 13), the University of Washington (n = 12), and the University of Miami (n = 11).

**Table 4:**
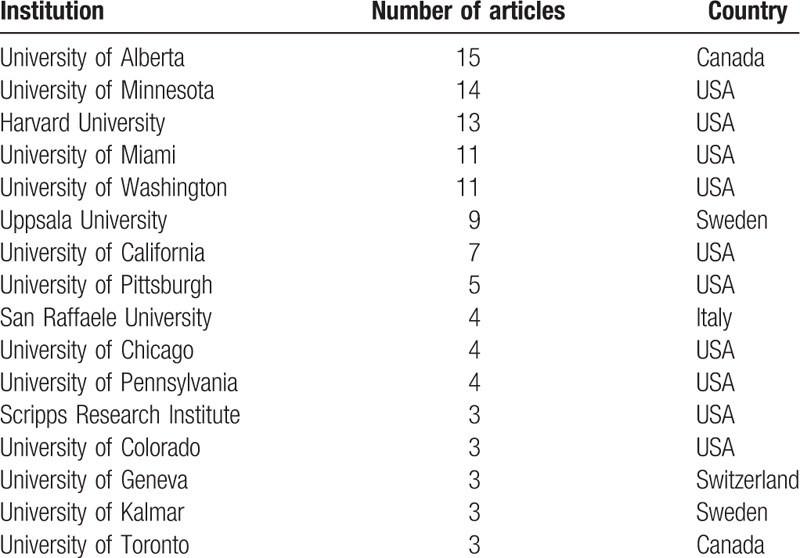
Institutions of origin with three or more top-cited articles in islet transplantation.

### 3.4 Journals

We found that the top-cited articles were published in 25 journals (Table 5). More than half (52%, n = 53) of the articles were published in journals that specialized in the fields of diabetes mellitus or transplantation; the largest number (n = 30) of articles was published in *Diabetes*. The remaining articles were published in less specialized journals; in particular, 2 general high-impact factor journals (*Proceedings of the National Academy of Sciences* [PNAS] and *Science*) published 9 and 8 articles, respectively (ranks 2 and 3). No correlations were found between the impact factors of the journals and the number of top-cited articles they published when the articles were stratified into 3 groups: a low impact factor (IF) group (0–4.9), a middle IF group (5–9.9), and a high IF group (≥10); the impact factors were, respectively, *r* = 0.11, *P* = .79; *r* = 0.37, *P* = .42; *r* *=* −0.26, and *P* = .46.

**Table 5:**
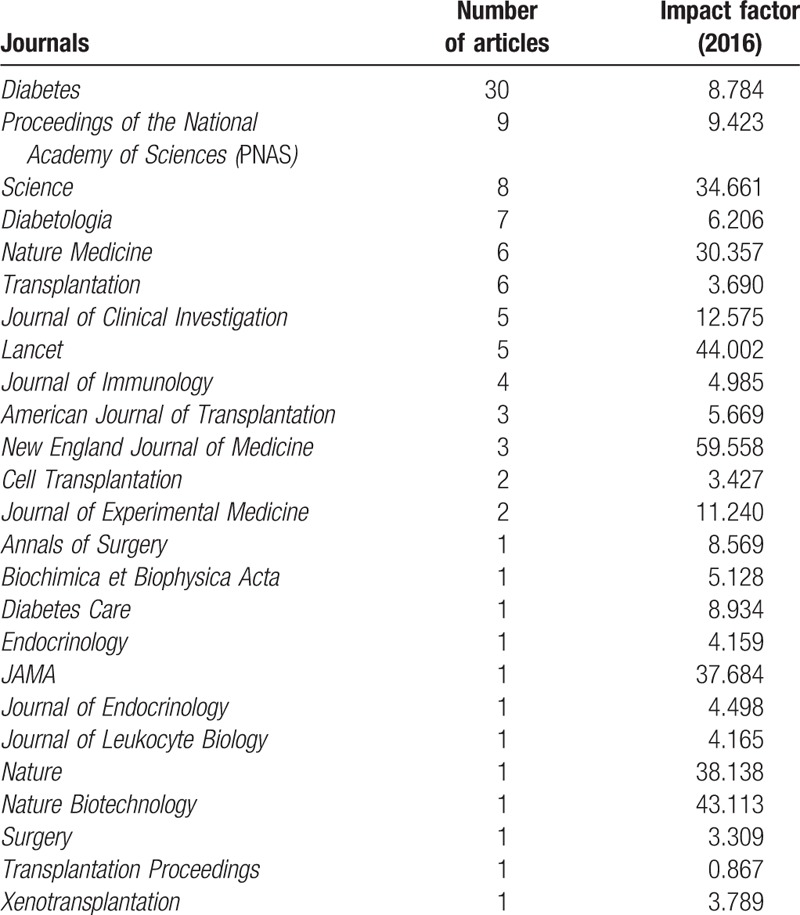
Journals publishing top-cited articles.

### 3.5 Investigation of possible factors influencing citations

**Figure 3:**
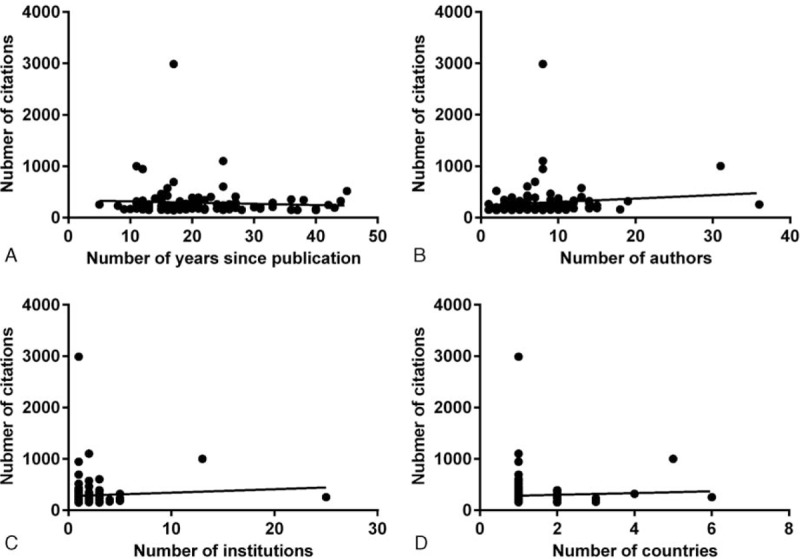
Correlations between the number of citations and the number of years since publication (A); the number of authors (B); the number of institutions (C); and the number of countries involved (D).

To identify the factors that determined the number of citations of top-cited articles, we investigated possible correlations between the number of citations and the number of authors, institutions, years since publication, and countries involved. No correlations were found between the number of citations and years since publication (*r* = 0.02, *P* = .79), authors (*r* = 0.13, *P* = .18), institutions (*r* = 0.08, *P* = .41), or countries involved (*r* = 0.02, *P* = .85) (Fig. 3).

### 3.6 Keyword analysis

**Figure 4:**
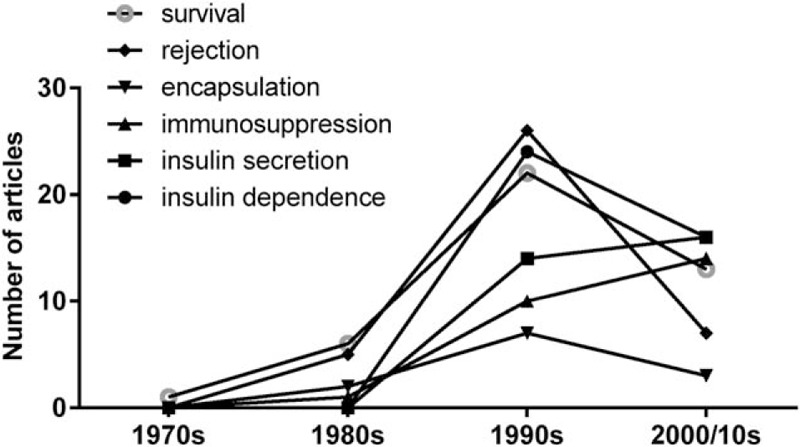
Trends over time: common keywords found in the 100 most frequently cited articles. 2000/2010: Only 1 article was written in the 2010; for this reason, the results from this decade were integrated into the 2000s.

**Figure 5:**
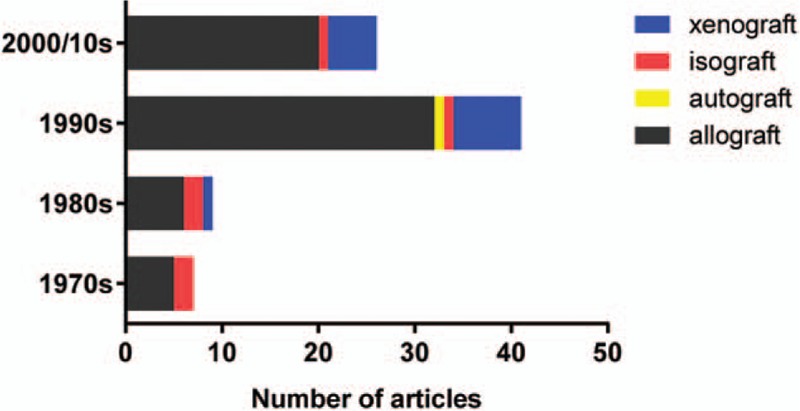
Time trends in the distribution of the 100 most frequently cited articles by graft type. 2000s/2010s: Only 1 article was written in the 2010; for this reason, the results from this decade were integrated into the 2000.

To assess the historical development of islet transplantation research and current trends, we analyzed variations in keywords from different periods. Overall, the most frequently used keywords were insulin dependence, insulin secretion, immunosuppression, encapsulation, rejection and survival. Keywords such as “insulin secretion” and “immunosuppression” increased in frequency between the 1970s and the 2000/10s, whereas keywords such as insulin dependence, rejection, survival, and encapsulation peaked during the 1990s. Only 1 article was from 2010, and so results from that decade were integrated into the 2000s (Fig. 4). Graft types were also top-listed keywords. The 100 most frequently cited articles were divided into 4 groups: allografts, autografts, isografts, and xenografts. In all decades, the allograft was the most common type of graft (76%), followed by the xenograft (16%); isografts and autografts were scare (Fig. 5).

### 3.7 Citation network

**Figure 6:**
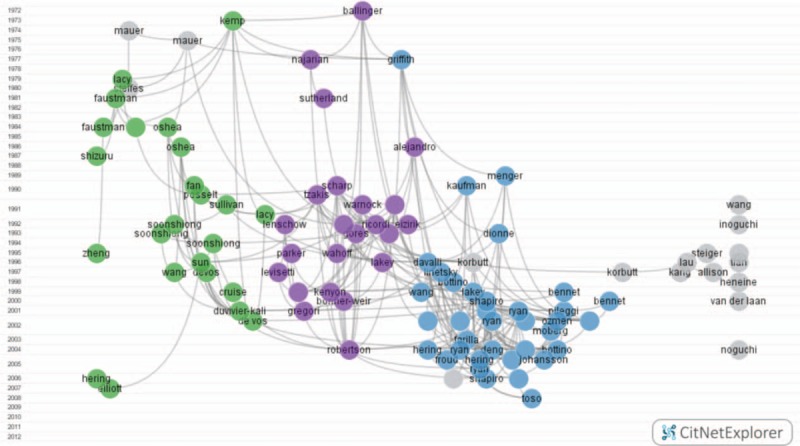
Citation networks visualizing the research topics of the 100 most frequently cited articles. Kemp-cluster: green; Ballinger-cluster: purple; Griffith-cluster: blue.

Citation network analysis was carried out to reveal the citation links and research topics of the 100 most frequently cited articles. Three close citation networks (3 clusters) were identified through a CitNetExplorer cluster analysis, as can be seen in Fig. 6. Three citation networks were rooted in 3 classical publications (Kemp CB, 1973; Ballinger WF, 1972; Griffith RC, 1977); we have therefore referred to them as the Kemp-cluster, the Ballinger-cluster, and the Griffith-cluster (Fig. 6). Publications included in the Kemp-cluster discussed the use of antirejection immunotherapies or biocompatible encapsulations to prolong graft survival. Publications included in the Ballinger-cluster tended to focus on assessing the efficacy (measured through insulin secretions and independence) of islet transplants. Publications in the Griffith-cluster discussed the structure and function of isolated or transplanted islets.

## 4 Discussion

In this study, we used bibliometric analysis to identify and characterize the 100 most frequently cited articles in the field of islet transplantation. The top-cited articles on islet transplantation were cited 146 to 2988 times. This number is much lower than the equivalent in some other medical fields, such as hypertension (582–7248) and diabetes (1121–10292), but higher than orthodontics (89–545) and orthopedic elbow surgery (124–388), to name only a few.^[12,14,15,25]^ Citations differed between specialties, mainly depending on the number of researchers in specific medical fields.^[26]^ Open access may be another important factor in attracting citations. Open-access (OA) articles are more easily recognized and cited than non-OA articles. ^[27]^ Further analysis has shown that, for the same author, publications in OA journals have a higher rate of citation than publications in traditional, non-OA journals in some medical fields, such as cytopathology. ^[28]^ However, OA articles have not received significantly more citations than non-OA articles in dentistry and ophthalmology. ^[29,30]^ It is therefore clear that the extent to which OA increases citations varies across different research fields.

The decade during which most top-cited articles in the field of islet transplantation were published was the 1990s. In all, 85 articles (83%) were published after 1990. This result suggests that it may take 15 years or more for article citations to peak, as documented through bibliometric analysis.^[25,31]^ There are several reasons for this finding. First, more articles on islet transplantation have emerged in the past 15 years, and researchers tend to cite the most recent study. In addition, Ricordi et al's^[32]^ discovery of an automated method for isolating islets from the human pancreas in 1989 greatly stimulated subsequent research into islet transplantation.

We have demonstrated that basic science is the most frequent type of study design. This trend runs counter to many medical specialties, in which most articles are observational studies.^[12,14,25,33]^ There is only 1 randomized clinical trial (level 1c) of islet transplantation; thus more high-level evidence of clinical trials were needed in the future research. Although the demand for islet transplantation among diabetic patients far exceeds the number of islets available, there are too few human pancreatic islets to meet the need for transplantation. Thus, the pressure to create an ample supply of islets has led to extensive basic-science research, including xenografts of porcine islets.^[4]^

Our study found that most top-cited articles were written in the US; 10 (62.5%) of the 16 top centers for islet transplantation are also located in the US. These results are in line with the origin of the 100 most frequently cited articles in many other medical fields.^[8,11,34]^ The US leads the world in medical research, given its large number of researchers and generous research funding. In addition, American authors tend to cite local articles and US reviewers prefer American articles.^[31,35,36]^

The 100 most frequently cited articles were published in 25 journals. Because the clinical application of islet transplantation is to treat diabetes mellitus, it is not surprising that more than half of the articles were published in journals specializing in the fields of diabetes mellitus or transplantation. As indicated by Bradford's law, a few core journals in the specialized field were mainly used to obtain citations; there were significantly fewer citations of articles published in non-core journals.^[37]^ Thus, most of top-cited articles appeared in a few major, specialized medical journals. In this case, the specialized journal *Diabetes* published the most articles (n = 30). However, many top-cited articles on islet transplantation were published in high impact factor general medical journals, such as *PNAS* (n = 9) and *Science* (n = 9).

Previous studies have shown that the impact factor of particular journals is the best indicator for citations; top-cited articles usually appeared in journals with high impact factors.^[38]^ Our study does not support this finding. Our results suggest that citations were not clearly influenced by the impact factor of the journals, as other reports have also shown.^[12]^ Perhaps, the tendency for top-cited articles to be published in general or specialized journals varies across different medical fields. Top-cited articles on hypertension and tuberculosis, for example, tend to be published in general journals, whereas those on lung transplantation are usually published in specialized journals.^[14,18,39]^ These findings suggest the need to develop measures to assess the significance of specific research papers that are more accurate.

Our study found a correlation between a high number of allografts and highly rated examples of islet transplantation, mainly because pancreatic islets are more readily available for allografts than for isografts or autografts. In addition, pancreatic islet allografts carried out using the Edmonton protocol result in successful insulin independence and glycemic control in type 1 diabetic patients.^[24]^ However, the clinical outcome of allogeneic islet transplantation is mainly complicated by graft rejection. An analysis of keywords and citation networks shows that many of the 100 most frequently cited articles discuss ways to prolong graft survival through antirejection immunotherapies or biocompatible encapsulations. It is postulated that, in future, research will focus on autografts that are not susceptible to immune rejection. Furthermore, the demand for islet transplantation will expand in patients with type 1 diabetes mellitus, causing the current supply of islets derived from human organ donors to fail to meet demand. Perhaps, porcine sources can provide sufficient islets to meet clinical demand, increasing xenograft research.

This study has some limitations. First, we have only used a single medical database, the SCIE, for our analysis. It is worth noting that the SCIE does not index all journals; as a result, some journals indexed by the Google Scholar or SCOPUS databases may have been missed. Although different databases return vastly different results,^[40,41]^ it is acceptable to use a single medical database to identify top-cited medical research articles—many published bibliometric analyses have used the SCIE database for this purpose. ^[14,20,42]^ Another potential source of bias involves the “obliteration by incorporation” effect described by Garfield et al, which has been demonstrated in the literature of other medical fields. ^[43,44]^ This effect refers to the phenomenon by which older classic articles gradually lose the spotlight position, and are no longer cited with the same frequency because their findings have been incorporated into the current knowledge of the field. To counter this effect, we used the total number of citations received, rather than the number of citations received during the current year to generate our list of top-cited articles. Using current-year citations as screening criteria would have caused an unfair relative increase in citations for more recent articles, overlooking many older classic articles. Using the total number of citations ensures that older articles, which have accumulated many citations over time, warrant inclusion.^[25]^ Other potential factors that affect citations cannot be accurately determined; these include journal and author self-citations; citations in textbooks, conferences, and web-based literatures; and omission bias.^[31,45]^ Such biases may affect the generation of a representative list of top-cited articles based on absolute citation counts.

## 5 Conclusions

Despite these limitations, this study successfully reveals the characteristics of top-cited articles in the field of islet transplantation. Most involve basic science, have been published in *Diabetes,* and originate from institutions in the US. Following on from clinically successful allografts using the Edmonton protocol, autografts and xenografts should be strengthened to solve the problems of immune rejection and islet sources, respectively.

## Acknowledgment

The authors would like to thank Bo Xu for his assistance and review of the manuscript.
